# Description and initial evaluation of incorporating electronic follow-up of study participants in a longstanding multisite cohort study

**DOI:** 10.1186/s12874-016-0226-z

**Published:** 2016-09-23

**Authors:** Kiarri N. Kershaw, Kiang Liu, David C. Goff, Donald M. Lloyd-Jones, Laura J. Rasmussen-Torvik, Jared P. Reis, Pamela J. Schreiner, Daniel B. Garside, Stephen Sidney

**Affiliations:** 1 Department of Preventive Medicine, Northwestern University Feinberg School of Medicine, 680 N Lake Shore, Suite 1400, Chicago, IL USA; 2 Department of Epidemiology, Colorado School of Public Health, 13001 E 17th Place, Aurora, CO USA; 3 Division of Cardiovascular Sciences, National Heart, Lung, and Blood Institute, 6701 Rockledge Drive, Suite 10197, Bethesda, MD USA; 4 Division of Epidemiology and Community Health, School of Public Health, 1300 S 2nd Street, Suite 300, University of Minnesota, Minneapolis, MN USA; 5 Department of Medicine, University of Illinois at Chicago, 1819 W Polk Street, Suite 246, Chicago, IL USA; 6 Division of Research, Kaiser Permanente Northern California, 2000 Broadway, Oakland, CA USA

**Keywords:** Pilot projects, Cohort studies, Epidemiology

## Abstract

**Background:**

The objective of this study was to evaluate a pilot program that allowed Chicago field center participants of the Coronary Artery Risk Development in Young Adults (CARDIA) study to submit follow-up information electronically (eCARDIA).

**Methods:**

Chicago field center participants who provided email addresses were invited to complete contact information and follow-up questionnaires on medical conditions electronically in 2012–2013. Sociodemographic characteristics were compared between those who did and did not complete follow-up electronically. The number of participant contacts by CARDIA staff needed before follow-up was completed was also evaluated.

**Results:**

Blacks and low socioeconomic position individuals were less likely to complete follow-up using the electronic questionnaire. Participants who used the electronic questionnaire for follow-up needed fewer contacts (e.g., median 1 contact compared with 3for contact information follow-up), but they also needed fewer contacts prior to eCARDIA (median 1 before and after eCARDIA).

**Conclusions:**

Findings suggest other approaches will be needed to maintain contact and elicit follow-up information from harder-to-reach individuals.

## Background

Loss to follow-up of research participants may bias longitudinal study findings due to differences in the characteristics of participants who drop out and those who continue to participate [1], making successful follow-up essential. One of the more commonly used strategies for maintaining high cohort retention is employing a systematic method for participant contact [2]. This typically involves phone or mail contact at regular intervals and making repeated attempts to obtain complete data. While this method can be highly effective, it can also be labor-intensive and quite costly, particularly for large studies. Given growing funding constraints [3], proven, effective alternative methods for participant follow-up are needed.

Electronic follow-up has the potential to lower costs by reducing the amount of time study staff members devote to contacting participants. It may also improve participant satisfaction by offering a convenient method for collecting follow-up information. However, it is not known how well participants will respond to electronic follow-up compared with more traditional forms of follow-up.

In this study, we summarize findings from the Coronary Artery Risk Development in Young Adults (CARDIA) study electronic follow-up pilot study (eCARDIA), in which participants at the Chicago field center were invited to complete follow-up information online. These findings will help shed some light on the utility of electronic follow-up in large prospective studies.

## Methods

### Study population

CARDIA is a prospective, multi-center investigation of cardiovascular disease risk factor trends and determinants in 5,115 Black and White men and women aged 18–30 years recruited in 1985–1986 and re-examined 2, 5, 7, 10, 15, 20, and 25 years later (2010–2011) [4, 5]. Participants are contacted twice a year between in-person examinations: once to update contact information and once for an update of contact information, hospitalizations, outpatient procedures, medical diagnoses, and medication use (medical conditions follow-up). The current study used follow-up information between 2012 and 2013 from the 1,023 Chicago field center participants who were alive, not lost to follow-up, and had not withdrawn from the study as of the year 25 examination.

### Follow-up data collection procedure

All participants were given the opportunity to provide their email addresses prior to the start of eCARDIA as part of the process of updating contact information between in-person examinations. Participants were emailed an invitation to complete follow-up 324, 330, and 336 months after baseline via electronic questionnaire. Electronic questionnaires were administered using the online software program SurveyGizmo (Widgix, LLC; Boulder, CO). Data collected at the 324 and 336 month follow-up included medical conditions follow-up. Electronic questionnaires were incorporated during the midst of the 324 month follow-up and thus not all participants with email addresses received an invitation to provide follow-up information electronically during this contact period. Only updated contact information data were collected at the 330 month follow-up; the electronic questionnaire was available to all participants beginning with this follow-up. Data from each participant’s first electronic contact information and medical conditions follow-ups (either at the 324 month or 336 month follow-up) are included in this study.

Email invitations were sent out on the 1st day of the month of each participant’s 6-month follow-up window. An initial reminder email was automatically sent to non-responders 2 weeks later, and a second reminder email was automatically sent out 2 weeks after that. Staff members contacted participants by telephone who did not respond to the email invitation after 6 weeks from the date the first email invitations were sent. During the telephone call, staff attempted to redirect participants to the electronic questionnaire, but they were also prepared to allow participants to complete the questionnaire over the phone. Participants without email addresses were followed through the standard routes, including telephone and regular mail.

### Study variables

Self-reported sociodemographic characteristics were collected at in-person examinations for CARDIA, and included race (black and white), education (≤12 years; 13–15 years; and ≥ 16 years), income (< $35,000; $35,000-$74,999; and ≥ $75,000), sex (male and female), and age (43–49 and 50–55). The number of contacts required for completion of follow-up was also measured, both just before and after the introduction of eCARDIA. These contacts reflect the number of times a staff member had to contact a participant before follow-up was completed, not automatic email reminders from SurveyGizmo.

### Statistical analysis

Sociodemographic characteristics (age, race, sex, income, and education) were compared descriptively between those who had email addresses on file and those who did not. All subsequent analyses were restricted to those with email addresses. Sociodemographic characteristics were compared between participants who completed follow-up via electronic questionnaire and those who did not using chi-square tests. The impact of the introduction of the electronic questionnaire on staff burden was evaluated in two ways. First, the number of participant contacts required to obtain participant follow-up information was compared between those who used the electronic questionnaire, those who completed follow-up but did not use the electronic questionnaire, and those who did not complete follow-up, both before and after the introduction of eCARDIA. Statistical significance (P < 0.05) was determined using the Kruskal-Wallis test. Second, the number of contacts before and after the introduction of eCARDIA were compared for each of the three groups. Statistical significance was determined using the Wilcoxon signed rank sum test. Analyses were conducted using SAS 9.4 (SAS Institute Inc., Cary, NC).

## Results

Approximately 68 % (n = 697) of Chicago field center CARDIA participants had email addresses on file. Participants with email addresses were more likely to be white (79.0 and 77.3 % of white men and women, respectively, compared with 50.0 and 61.7 % of black men and women). They were also more likely to have high annual incomes (87.1 % of participants with annual incomes ≥ $75,000 compared with 44.9 % of those with incomes < $35,000) and levels of educational attainment (82.6 % among those with ≥ 16 years of education compared with 42.3 % among those with ≤12 years).

**Fig. 1:**
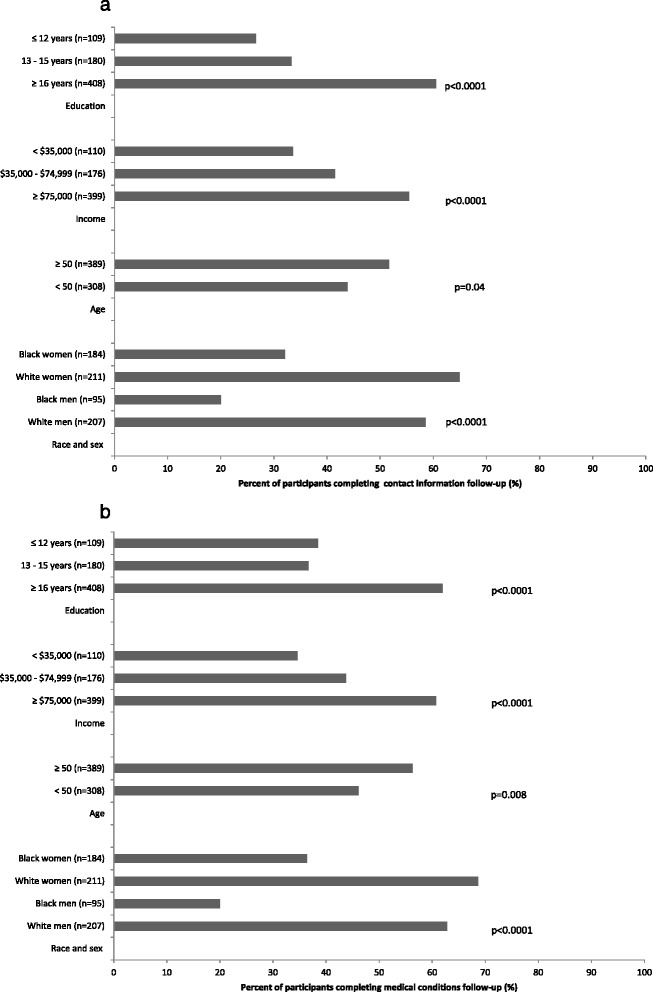
Percentage of CARDIA participants at the Chicago field center with email addresses who used the electronic questionnaire to update (**a**) contact information and (**b**) medical conditions

Among CARDIA participants with email addresses, those who completed contact information follow-up using the electronic questionnaire were more likely to be in the highest income and education categories (Fig. 1a). They were also more likely to be ≥50 years old and to be white. Findings were similar in direction and stronger in magnitude for the medical conditions follow-up (Fig. 1b).

Table 1 shows the median number and interquartile range of contacts required for completion of follow-up among those who did and did not use the electronic questionnaire for follow-up. We found that both before and during eCARDIA, those who completed follow-up using the electronic questionnaire required fewer contacts than those who completed follow-up using other modes and those who did not complete follow-up. Participants who completed contact information follow-up via electronic questionnaire required somewhat fewer contacts from CARDIA staff than they did prior to eCARDIA (interquartile range from 1 – 2 vs. 1 – 3 before eCARDIA). Approximately 57.7 % of electronic questionnaire users completed contact information follow-up after the initial request from CARDIA staff before eCARDIA compared with 71.1 % during eCARDIA (not shown). There was no statistically significant difference in the number of contacts needed to complete medical conditions follow-up among electronic questionnaire users.

**Table 1:** Median (interquartile range; IQR) number of contacts required for completion of follow-up by use of electronic questionnaire and type of follow-up among CARDIA participants at the Chicago field center

	Last follow-up before eCARDIA pilot	Follow-up during eCARDIA pilot	*P*-value^**^
	Median (IQR)	Median (IQR)	
Contact information follow-up*			
Follow-up completed using electronic questionnaire (n = 336)	1 (1 – 3)	1 (1 – 2)	0.0002
People who completed follow-up using another mode (n = 341)	3 (1 – 4)	3 (2 – 4)	0.0009
People who did not complete follow-up (n = 20)	4 (3 – 6)	8 (7 – 9)	0.004
Medical conditions follow-up*			
Follow-up completed using electronic questionnaire (n = 361)	1 (1 – 3)	2 (1 – 3)	0.47
People who completed follow-up using another mode (n = 302)	3 (1 – 5)	4 (2 – 5)	0.0001
People who did not complete follow-up (n = 34)	6 (3 – 11)	9 (7 – 12)	0.02

^*^ *P* < 0.0001 comparing the three categories of follow-up based on the Kruskal-Wallis test

^**^ *P*-value comparing participant contacts before and after eCARDIA based on the Wilcoxon signed rank sum test

## Discussion

We found that email address availability and electronic questionnaire utilization were highly patterned by race and socioeconomic position. Possible alternatives to engage minority and participants of low socioeconomic position may be text messaging or using smart phone applications. A 2014 survey conducted by the Pew Research Center found that 90 % of all adults have a cellphone, including 90 % of African-Americans, 87 % of those with a high school diploma or less, and 84 % of those with annual household incomes below $30,000 [6]. In addition, 59 % of African-Americans, 44 % of those with a high school diploma or less, and 47 % of those with household incomes below $30,000 owned smartphones. These latter percentages are comparable to the percent of participants with email addresses in CARDIA (60.0 of blacks, 47.4 of those with ≤ 12 years of education, and 46.2 % with annual household income < $35,000; not shown in tables). Few studies have examined the effectiveness of technology use in healthcare settings among minority and/or low-income populations, but there is some evidence suggesting text messaging interventions improve health behaviors and disease management [7, 8]. Future work is needed to determine whether text messaging or a smartphone application may improve contact with harder-to-reach participants.

We also found that CARDIA participants who used the electronic questionnaire were generally better responders, and that offering the electronic questionnaire only slightly reduced the required number of staff contacts in this group for contact information follow-up and did not reduce the number of staff contacts at all for the medical conditions follow-up. This suggests offering the electronic questionnaire may serve as a convenient option for easy-to-reach participants and may even reduce the already low staff burden required to maintain contact with these participants. However, the electronic questionnaire did not ease staff burden for those who already required more contacts to complete follow-up.

## Conclusions

In summary, these findings indicate more work is needed to determine how best to use electronic follow-up to reduce staff burden and reduce costs associated with following a large sample of participants in a long-term population-based study. Future research exploring other forms of electronic follow-up, particularly approaches that take advantage of mobile technologies, may represent promising alternatives or complements to email follow-up. In the meantime, staff contact via telephone remains a necessary mode of follow-up to ensure the valuable racial and socioeconomic diversity offered by these cohorts is maintained.

## Abbreviations

CARDIA: Coronary Artery Risk Development in Young Adults
